# Deciphering the conserved catalytic step of PAL-driven salicylic acid biosynthesis pathway in plants

**DOI:** 10.1093/hr/uhaf255

**Published:** 2025-09-17

**Authors:** Qian Hu, Gaofeng Liu, Zixin Zhang

**Affiliations:** College of Horticulture and Landscape Architecture, Southwest University, Chongqing 400715, China; College of Horticulture and Landscape Architecture, Southwest University, Chongqing 400715, China; College of Horticulture and Landscape Architecture, Southwest University, Chongqing 400715, China

Salicylic acid (SA), a pivotal phenolic hormone implicated in plant defense mechanisms, was first isolated from willow bark. It has attracted considerable attention for its capacity to initiate immune responses in plants when they are attacked by pathogens [1–3]. The biosynthesis of SA in plants originates from chorismate and primarily occurs via two distinct pathways: the isochorismate synthase (ICS) pathway and the phenylalanine ammonia lyase (PAL) pathway [4]. Studies reveal that *Arabidopsis thaliana* (Arabidopsis) sustains constitutively low SA levels, while pathogen or stress triggers activate SA biosynthesis predominantly via the ICS-dependent pathway [5]. Extensive studies on most members of the *Brassicaceae* family have confirmed the functional conservation of this pathway [6]. Notably, while ICS-encoding genes are widely distributed across plants, no changes in SA content were detected in the *ICS*-deficient mutants of non-*Brassicaceae* species (rice, barley, wheat) [7–9]. It is suggested that the ICS-mediated pathway may have evolved relatively recently in *Brassicaceae* family and is unlikely to represent the primary route for SA biosynthesis in most plant species [1, 7]. Current evidence points to PAL-mediated pathways as the principal SA synthesis routes beyond *Brassicaceae*, though mechanistic details remain poorly characterized. Earlier hypotheses posited benzoic acid 2-hydroxylase (BA2H) as a putative catalyst converting benzoic acid to SA, yet the molecular identity of BA2H remains elusive [10]. Crucially, emerging evidence now challenges this premise, demonstrating that SA biosynthesis is not from phenylalanine but from benzoic acid in Arabidopsis [3].

**Figure 1:**
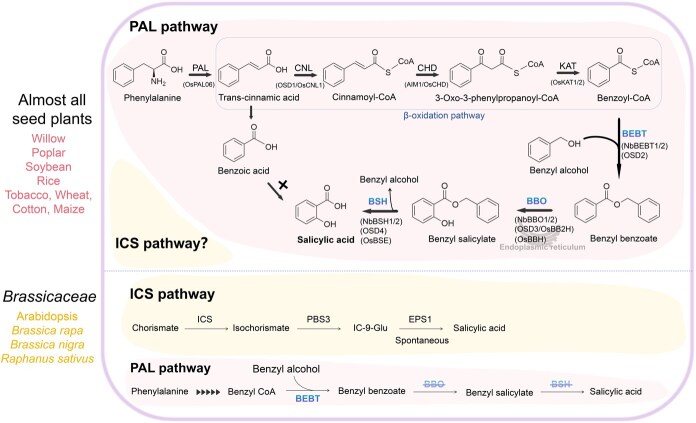
SA biosynthesis in seed plants. Unlike the ICS pathway that is mainly used in *Brassicaceae*, the new identified PAL pathway appears to be commonly used in plants. Genes encoding the homologs with high similarity to *NbBEBT*, *NbBBO* and *NbBSH* are present in a broad range of plants, except for Arabidopsis, *Brassica rapa*, *Brassica nigra* and *Raphanus sativus* in the *Brassicaceae* family, which only possess homologs exhibiting high similarity to *NbBEBT*. Functional complementation assays were systematically performed across dicots such as willow, poplar and soybean as well as the monocot rice, confirming the pathway’s universality, which can complement the phenotype of SA-deficient mutants of *Nicotiana benthamiana*. The full characterization of the PAL-SA pathway in rice through functional analysis of the peroxisomal OSD2, endoplasmic reticulum-resident OSD3, and cytoplasmic OSD4 enzymes. Benzoic acid is not a direct biosynthetic precursor of SA in established model systems. The β-oxidation pathway, has been shown to convert trans-cinnamic acid to benzoyl-CoA, mediated by enzymes such as OSD1/cinnamate-CoA ligase (OsCNL), Abnormal inflorescence meristem1 (AIM1)/cinnamoyl-CoA hydratase/dehydrogenase (OsCHD) and 3-ketoacyl-CoA thiolase (OsKAT1/2) in rice, and the NbCNL-NbCHD-NbKAT module in *Nicotiana benthamiana*. In the *Brassicaceae* family, SA biosynthesis proceeds mainly through the ICS-mediated pathway, a spatially separated pathway across chloroplasts and the cytoplasmis, which ICS catalyzes the conversion of chorismate (CA) into isochorismate (IC) within the chloroplast. It is subsequently transported to the cytoplasm, where PBS3 facilitates the transformation of IC into isochorismate-9-glucoside (IC-9-Glu). EPS1 catalyzes the last step, converting IC-9-Glu into SA.

In a groundbreaking work published in the latest *Nature* issue, Liu *et al.* [1] have unveiled a novel benzoyl-CoA-dependent three-step biosynthesis of SA in plants, which resolves the perplexity by demonstrating that SA is synthesized via an alternative route in most seed plants (Fig. 1). Concurrently, in companion studies [2, 3] published in tandem with this work, the rice PAL pathway for SA biosynthesis (PAL-SA pathway) was also elucidated. These two seminal articles collectively offer interlocking validation through phylogenetic and functional complementation analyses, while establishing an evolutionarily conserved core module for SA biosynthesis. Together, they mark a paradigm-shifting advance in research on plant specialized metabolism, resolving decades-long ambiguities concerning the SA biosynthesis pathway. The latest research Liu *et al.* [1] systematically demonstrated pathogen-induced pathway specialization: *Pseudomonas syringae* pv. *tomato* (*Pst*) *DC3000* infection triggered coordinated upregulation of multiple *PAL* genes concurrent with suppression of two *ICS* homologs, strongly implicating PAL-mediated rather than ICS-dependent SA biosynthesis. Transient overexpression of hairpin RNAs (hp-RNAs) that specifically target *PAL* genes, but not *ICS* genes, led to a significant reduction in SA accumulation, confirming pathway divergence. Then, an ethyl methane sulfonate mutagenesis screen was conducted to isolate nine *Pst DC3000*-insensitive loss-of-function mutants, thereby facilitating the identification of key regulators of this pathway. Combined genetic mapping and positional cloning revealed that a mutation in *NbL18g16990* caused SA deficiency. This gene encodes a benzoyl-CoA transferase (BEBT), confirming its essential role in SA biosynthesis. Utilizing *Nicotiana benthamiana* as a model, the authors identified benzoyl-CoA and benzyl alcohol as substrates for BEBT, catalyzing the formation of benzyl benzoate (BB) through esterification. Subsequent hydroxylated by benzyl benzoate oxidase (BBO) to benzyl salicylate (BS), and finally, hydrolysis of BS leads to SA production (Fig. 1). Complementation experiments using orthologs from dicots, including willows, poplars, and soybeans, as well as monocots, such as rice, confirming the pathway’s universality. Enzymes from diverse species have been shown to rescue SA-deficient phenotypes in *N. benthamiana*. Notably, the *Brassicaceae* family (e.g. Arabidopsis, *Brassica rapa*, *B. nigra*, *Raphanus sativus*) lacks functional BBO and BS hydrolase (BSH) homologs, which explains their reliance on the ICS pathway [1]. This conservation highlights the evolutionary adaptability of the BEBT-BBO-BSH pathway and suggests its primacy in non-*Brassicaceae* species. Moreover, the involvement of peroxisomal β-oxidation in generating benzoyl-CoA (via cinnamic acid) links SA biosynthesis to lipid metabolism, opening new avenues for metabolic engineering to optimize SA production. In another concurrent studies in *Nature*, Zhu *et al.* [2] and Wang *et al.* [3] identified the PAL-SA pathway in rice. Comprehensive biochemical and genetic evidence unequivocally establishes the SA biosynthetic pathway in rice as a three-step enzymatic cascade: benzoyl-CoA → BB → BS → SA (Fig. 1). This sequential transformation is mediated by *Oryza sativa SA-Deficient gene 2* (*OSD2,* homolog of *BEBT*); *OSD3/OsBB2H, benzyl benzoate 2-hydroxylase* (homolog of *BBO/BBH, benzylbenzoate hydroxylase*); and *OSD4* (homolog of *BSH/BSE, benzylbenzoate hydroxylase*)–with subcellular fractionation confirming their cellular compartmentalization during catalysis [2, 3]. Subsequently, through an evolutionary analysis [2] and the key enzymes respectively identified [2, 3] as being involved in SA biosynthesis, it was revealed that the PAL-SA pathway emerged prior to the divergence of gymnosperms and has been conserved in most seed plants. And this finding was also corroborated by isotope tracing experiments.

By establishing a conserved framework for SA production in seed plants, these three works not only resolve long-standing controversies but also pave the way for precision engineering of plant immunity. Genetic studies have revealed that the ICS-SA pathway is absent in tobacco and rice plants [1, 7]. And whether the residual SA is derived from the ICS-mediated pathway remains to be elucidated. Although the ICS pathway is proposed as *Brassicaceae*-specific, this hypothesis requires validation in additional plant species by experiments. Similarly, in the *Brassicaceae* family, further exploration of potential pathways other than the main ICS-dependent SA synthesis pathway that may lead to the accumulation of residual SA is highly instructive and will add a significant stroke to the genetic evolution of the species. Both studies employed forward genetic screening, genetic evolutionary analysis, and functional validation to identify the conserved PAL–SA biosynthetic pathway in most seed plants (Fig. 1). Liu *et al.*’s research [1] initiated from enzymes identified in *N. benthamiana* and performed genetic transformation verification and evolutionary analysis across multiple species, demonstrating the high conservation of the three-step SA biosynthesis pathway originating from benzoyl-CoA in seed plants outside the *Brassicaceae* family. Zhu *et al.*’s study [2] focused on rice and identified key genes (*OSD1*–*OSD4*) involved in the PAL-SA biosynthetic pathway from phenylalanine to SA, elucidating their biochemical functions and subcellular localization. Wang *et al.*’s complementary [3] *in vivo* isotope labeling and enzyme reconstitution experiments established the sequential action of BBH and BSE in converting benzylbenzoate to SA through the benzylsalicylate intermediate. Meanwhile, evolutionary analyses revealed that the PAL–SA pathway emerged prior to the divergence of gymnosperms and has been conserved in most crops, such as maize, wheat, and cotton. Furthermore, Zhu *et al.*’s study [2] demonstrated that activating this pathway (e.g. overexpression of *OSD1*) significantly enhances SA levels and pathogen resistance in rice, providing new targets for crop disease-resistance breeding.

The identification of SA synthetic pathway provides a robust and reliable foundation for the subsequent clarification of how pathogen-induced signal transduction regulates the activity of the BEBT-BBO-BSH pathway. Continued exploration of the compartmentalized storage and transport mechanisms of SA within cells is of great significance, particularly in elucidating how cytotoxicity can be avoided at elevated SA levels. Future studies integrating systems biology and synthetic biology approaches will undoubtedly unlock the full agronomic potential of this ancient metabolic pathway.
